# Extracellular Matrix Degradation and Tissue Remodeling in Periprosthetic Loosening and Osteolysis: Focus on Matrix Metalloproteinases, Their Endogenous Tissue Inhibitors, and the Proteasome

**DOI:** 10.1155/2013/230805

**Published:** 2013-06-19

**Authors:** Spyros A. Syggelos, Alexios J. Aletras, Ioanna Smirlaki, Spyros S. Skandalis

**Affiliations:** ^1^Department of Anatomy, Histology, Embryology, Medical School, University of Patras, 26500 Patras, Greece; ^2^Laboratory of Biochemistry, Department of Chemistry, University of Patras, 26500 Patras, Greece

## Abstract

The leading complication of total joint replacement is periprosthetic osteolysis, which often results in aseptic loosening of the implant, leading to revision surgery. Extracellular matrix degradation and connective tissue remodeling around implants have been considered as major biological events in the periprosthetic loosening. Critical mediators of wear particle-induced inflammatory osteolysis released by periprosthetic synovial cells (mainly macrophages) are inflammatory cytokines, chemokines, and proteolytic enzymes, mainly matrix metalloproteinases (MMPs). Numerous studies reveal a strong interdependence of MMP expression and activity with the molecular mechanisms that control the composition and turnover of periprosthetic matrices. MMPs can either actively modulate or be modulated by the molecular mechanisms that determine the debris-induced remodeling of the periprosthetic microenvironment. In the present study, the molecular mechanisms that control the composition, turnover, and activity of matrix macromolecules within the periprosthetic microenvironment exposed to wear debris are summarized and presented. Special emphasis is given to MMPs and their endogenous tissue inhibitors (TIMPs), as well as to the proteasome pathway, which appears to be an elegant molecular regulator of specific matrix macromolecules (including specific MMPs and TIMPs). Furthermore, strong rationale for potential clinical applications of the described molecular mechanisms to the treatment of periprosthetic loosening and osteolysis is provided.

## 1. Pathobiology of Periprosthetic Loosening Process

 The total hip or knee replacement is an operation whereby the damaged cartilage and the subchondral sclerotic bone of the hip or knee joint are surgically replaced with artificial materials. The continuous improvement of the materials and the surgical techniques have given comfort to patients suffering from painful diseases of the joints, such as primary osteoarthritis and secondary ones caused by rheumatoid arthritis, posttraumatic conditions, congenital dysplasia or dislocation, and aseptic necrosis of the femoral head. After the improvement in prophylaxis against infection, aseptic loosening of endoprostheses represents the predominant complication of this operation, which usually occurs during the second decade, after the primary arthroplasty. Although many reports have been published on the pathogenesis of periprosthetic loosening, the precise biological mechanisms responsible for this process have not yet been completely elucidated. 

 Wear-generated particular debris at the interface between implant components is associated with chronic inflammation and osteolysis, limits the lifespan of the implants, and is the main cause of initiating this destructive process. However, many other factors, such as cyclic loading or micromotion of the implants and hydrostatic fluid pressure, have also been implicated revealing the high heterogeneity in the histology of the tissue around the prosthesis [1]. Evidence in support of the central role of wear debris in periprosthetic loosening and osteolysis includes the observations that osteolysis is correlated with higher wear rates [2] and that vast numbers of wear particles are found associated with the periprosthetic interfacial membrane removed during revision surgery [3–5]. Furthermore, experimental systems have demonstrated that particulate debris can induce osteolysis in a variety of animal models [6–12] and inflammatory responses in cultured macrophages [8, 13–17]. Wear debris may include particles from all the various components of the prosthesis (such as polyethylene, metal, and ceramic) as well as bone cement [18]. Since cellular responses are highly dependent upon the composition, size, and shape of particles, the type of prosthesis and bearing surface used may have a significant impact on the potential for development of osteolysis [19].

**Figure 1:**
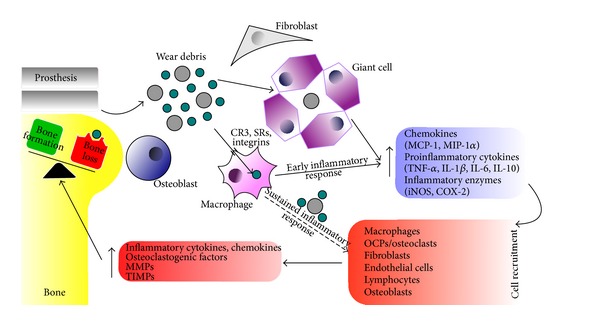
Schematic representation of periprosthetic loosening and osteolysis. Implant-derived wear debris induces an early inflammatory response from the resident or infiltrating macrophages in the periprosthetic tissue. Small particles are phagocytosed, whereas the larger induce fusion of macrophages and giant cell formation. Activated macrophages release proinflammatory cytokines, chemokines, and enzymes that recruit multiple cell types within periprosthetic tissue, which are further activated by the particles resulting in sustained inflammation, increased secretion of cytokines/chemokines/osteoclastogenic factors/MMPs/TIMPs, and osteolysis.

 The release of implant-derived particles induces a cellular host response, which initially is taking place in the pseudocapsular tissue (PCT). This membranous tissue is formed postoperatively around the artificial joint and practically replaces the normal joint capsular tissue, which is usually removed during the primary joint replacement procedure. The most important and active cells in this tissue are macrophages and fibroblasts, which after their interaction with the wear debris produce most of the soluble chemical factors and mediators, which are going to be analyzed below. Additionally these soluble factors migrate through the joint fluid (pseudosynovial fluid, PSSF) in the layer between the implant and the bone (interface), where they continue their action, mainly affecting the bony tissue. Finally the fibrous interface tissue (IFT), between the prosthesis and the bone, is formed and this leads to failure of the implant, which becomes loose. The communication of the interface layer with the space of the initial foreign body reaction is described as effective joint space, may result an early micromotion of the implant, and could be related to the surgical technique [20]. The interface tissue is heavily infiltrated with several different cell types, mainly macrophages, lymphocytes, fibroblasts, endothelial cells, and osteoclast precursors (OCPs)/osteoclasts. Beside enhanced and chronic inflammatory reactions in the periprosthetic region, the cellular recruitment to this region is promoted by induced chemokine expression [21–25]. Macrophages activation by phagocytosis of the wear debris particles, which are impervious to enzymatic degradation, has been shown to be the principle pathophysiologic mechanism in particle-induced periprosthetic osteolysis. Activated macrophages secrete proinflammatory and osteoclastogenic cytokines as well as proteolytic enzymes exacerbating the inflammatory response leading to activation of a periprosthetic osteolytic cascade (Figure 1). It is known that particles smaller than 8–10 *μ*m are phagocytosed by macrophages, while bigger particles induce giant cell reaction and are associated with such cells [26]. However, it has been reported that contact between wear particles and macrophages without phagocytosis is also important for the signal transduction of cytokines and activation of macrophages [14].

 The identity of the macrophage surface receptors responsible for recognition of the particles and the full repertoire of signaling cascades initiated or modified by particle binding remain poorly understood although macrophages are the best-characterized cellular target for particle action. One important consideration in determining the involvement of specific cell surface receptors is the extent to which different particles become opsonized with host serum proteins prior to phagocytosis. There is evidence that polyethylene activates complement [27], and this would argue in favor of a role for complement receptors, such as complement receptor 3 (CR3), in particle uptake. Indeed, CR3 expressing phagocytes have been detected in granulomatous lesions associated with hip replacement [28]. An involvement of CR3 in particle action is also supported by observations that antibodies against CR3 reduce particle uptake [14] and that activation of this receptor mimics several aspects of downstream signaling by particles in that MAP kinases [29] and the transcription factors nuclear factor *κ*B (NF-*κ*B) and activator protein-1 (AP-1) are activated [14, 30], and production of proinflammatory cytokines [14] and chemokines [30] is elevated. By contrast, research on alveolar macrophage response to environmental particulate matter has implicated scavenger receptors (SRs), such as scavenger receptor A (macrophage receptor with collagenous structure; MARCO), in opsonin-independent uptake of titanium particles [31], suggesting that different particles may use different surface receptors. Accordingly, Rakshit and coworkers suggested the involvement of opsonization, complement, and integrin receptors, including CR3 and fibronectin receptors, in polymethylmethacrylate action, and an involvement of scavenger receptors (scavenger receptor A) in macrophages responses to titanium [32]. This would provide an intriguing explanation of the abilities of different types of wear debris to elicit particle type-specific responses in cultured macrophages. The concept that opsonization may differentially regulate uptake of different compositions of wear debris is also supported by observations that the spectra of adherent human serum proteins demonstrate a level of particle specificity [33]. 

Other cell types that are abundant within the periprosthetic tissue are fibroblasts and osteoclasts. Frequently, a proliferation of periprosthetic fibroblasts, which constitute a major source of proinflammatory and osteoclastogenic mediators [34–37], is accompanied by tissue hypervascularization. Periprosthetic fibroblasts exposed to wear and/or proinflammatory mediators are a major source of the receptor activator for nuclear factor *κ*B ligand (RANKL) required to drive osteoclastogenesis in patients with osteolysis (discussed below). Particles can also induce production in cultured fibroblasts of proinflammatory mediators, collagenases, and stromelysins [36, 37], which contribute to the development of osteolysis and chemokines, which promote the recruitment of increased numbers of osteoclast precursors to periprosthetic tissues. The final cellular consequence of particle action is an excess of osteoclast activity, which results in uncontrolled bone erosion. Osteoclasts, which are the unique cell type capable to resorb bone, are derived from circulating hematopoietic cells of the monocyte/macrophage lineage. Therefore, wear particles might increase osteoclast activity either by generation of functional osteoclasts from osteoclast precursor cells within the periprosthetic space or recruitment of osteoclast precursor cells from the blood or both [19]. However, it is not only an increased osteoclastic bone resorption due to particle exposure that can disrupt the balance in the bone remodeling process, but also a reduced bone formation caused by a direct negative impact of particles on osteoblasts [38]. As shown by Lochner and coworkers, wear particles can alter the metabolism of human primary osteoblasts [39]. In particular, metallic particles in the wear debris of cemented hip endoprostheses can compromise the vitality and activity of bone cells and bone matrix. In consequence, this may lead to a reduction of implant integration strength. Osteoblasts are rather responsible for bone formation but can indirectly participate in bone degeneration by changing cell viability and expression of specific chemokines as well as directly by the secretion of preosteolytic mediators and specific proteinases.

 Collectively, the extensive body of research on *in vitro* cellular responses to wear debris suggests that while an inflammatory response by macrophages is central to the development of periprosthetic osteolysis, the detailed nature of this response will vary based upon several parameters, including prosthetic type, patterns of wear, cellular cross-talk, host factors, and cell-associated/extracellular molecular effectors.

## 2. Matrix Metalloproteinases, Their Endogenous Tissue Inhibitors, and Cytokines/Chemokines in the Periprosthetic Extracellular Matrix 

 Extracellular matrix (ECM) degradation and connective tissue remodeling around implants have been considered as major biological events in the periprosthetic loosening. Critical mediators of wear particle-induced inflammatory osteolysis released by periprosthetic synovial cells (mainly macrophages) are inflammatory cytokines (such as tumor necrosis factor-*α* (TNF-*α*), interleukin- (IL-) 1*β*, IL-6, and IL-10), chemokines (monocyte chemoattractant protein-1 (MCP-1), macrophage inflammatory protein-1alpha (MIP-1*α*)), inflammatory enzymes (inducible nitric oxide synthase (iNOS), cyclooxygenase-2 (COX-2)), and proteolytic enzymes, mainly matrix metalloproteinases (MMPs). 

 TNF-*α*, IL-1*β*, and IL-6 are known to be important molecules involved in the foreign body reaction process, and their upregulation is considered to be a marker of inflammation. They are well recognized as key proinflammatory cytokines that provoke cellular proliferation, stimulate osteoclast formation, and increase bone resorption around prostheses [40–44]. In particular, TNF-*α* has a catabolic effect on bone. It can upregulate bone resorption in cultured mouse calvaria by a prostaglandin-independent mechanism and stimulates osteoblasts to produce osteoresorptive factors such as IL-6 and prostaglandin E_2_ (PGE_2_) [45, 46]. High levels of TNF-*α* have been detected in periprosthetic tissues of loose endoprostheses with focal osteolysis [47]. It has also been shown to exhibit a synergistic effect with titanium particles, when added in osteoblast culture [48]. IL-1*β* induces differentiation and proliferation of osteoclasts as well as the production of MMPs and PGE_2_ from fibroblasts and synovial cells [49, 50]. It also reduces the osteocalcin production by the osteoblasts [51]. According to Jiranek and coworkers, IL-1*β* might play a significant role in the formation of IFT, because of its stimulatory activity on fibroblasts [52]. Kusano and coworkers have shown that IL-1*β* augments bone resorption in mouse calvaria culture *in vitro*, by inducing MMP-2, MMP-3, MMP-9, and MMP-13 production [53]. IL-6 is strongly implicated in bone catabolism. It is produced by the osteoblasts and induces bone resorption [54]. It also stimulates the formation of osteoclast-like cells in long-term human marrow cultures [55]. In periprosthetic tissues from loose orthopaedic implants with osteolysis, IL-6 levels are much higher than in tissues from loose implants without bone loss [47]. The role of prostaglandins in mediating pseudomembrane-associated bone resorption remains questionable. It is proposed, from *in vitro* studies, that prostaglandins play an important role in bone resorption [56]. Periprosthetic tissue, cultured in the presence of indomethacin, showed less bone resorptive capacity. Other investigators have shown that conditioned media from predialysed periprosthetic tissue cultures maintained their ability to cause bone resorption, indicating that the prostaglandins, removed by dialysis, had no effect whatsoever upon bone resorption [57]. Therefore, prostaglandins may be implicated in the loosening process through complex mechanisms involving interactions with MMPs and cytokines. On the other hand, IL-10 is synthesized by activated immune cells, in particular monocytes/macrophages, and has profound anti-inflammatory and immunoregulatory effects. This anti-inflammatory cytokine diminishes the expression of inflammatory mediators, inhibits antigen presentation, and induces expression of endogenous TNF-*α* inhibitors (soluble TNF receptors) to suppress the effects of proinflammatory cytokines in periprosthetic tissues [47, 58]. 

 Chemokines play pivotal roles in the recruitment of inflammatory and immune cells subsequent to the development of periprosthetic inflammation following wear particle generation. MCP-1 and MIP-1*α* are two chemokines involved in this adverse process by recruiting monocytes/macrophages and lymphocytes to the site around prostheses and play important roles in periprosthetic osteolysis [59–61]. Previous studies suggest that high levels of inflammatory enzymes, such as iNOS and COX-2, are also present in the tissues around prostheses and therefore may account for periprosthetic bone resorption [62]. Macrophages are the major inflammatory cells accounting for this response. iNOS is closely involved in regulating inflammatory responses and COX-2 is induced by many cytokines, such as TNF-*α* and IL-1*β*, and the overexpression of these two enzymes plays a key role in chronic inflammatory diseases [63]. Furthermore, iNOS and COX-2, as well as TNF-*α* and IL-6, are inductive regulators of osteoclastogenesis [64].

 A key role in periprosthetic ECM remodeling and destruction belongs to MMPs because of their ability to degrade in concert most extracellular matrix components, such as collagens, gelatin, elastin, laminin, fibronectin, or proteoglycan core proteins. MMPs contain four well-defined domains: a signal peptide, a propeptide with a conserved cysteine residue, a catalytic domain with a Zn-binding site, and a hemopexin-like domain at the COOH-terminal region, and they are frequently subgrouped based on substrate specificities and sequence characteristics. There are six main families of MMPs: collagenases (MMP-1, MMP-8, and MMP-13), gelatinases (MMP-2 and MMP-9), stromelysins (MMP-3, MMP-10, and MMP-11), matrilysins (MMP-7 and MMP-26), membrane-type MMPs (MT-MMPs: MMP-14, -15, -16, -17, -24, and -25), and other MMPs, which are not categorized in any of the previous groups (MMP-12, -19, -20, -21, -23, -27, and -28). The expression of MMPs is under tight control at the transcription level and their proteolytic activity is regulated posttranslationally in several ways [65]. MMPs are synthesized as zymogens, which are then activated extracellularly, with the exception of MMP-11 (stromelysin 3), MT-MMPs, MMP-21, MMP-23, and MMP-28. Although pro-MMPs can be activated *in vitro* by various proteolytic and nonproteolytic means, the *in vivo* activation mechanisms have not yet been completely clarified. Further, the proteolytic activity of MMPs is regulated by specific tissue inhibitors of metalloproteinases (TIMPs). Four TIMPs have been identified (named TIMP-1 to -4), which form high-affinity 1 : 1 noncovalent complexes with all active MMPs, thereby inhibiting their action. TIMPs inhibit all MMPs tested so far, but TIMP-1 is a poor inhibitor for MT3-MMP, MT5-MMP, and MMP-19. TIMP-3 has been shown to inhibit members of the ADAM (a disintegrin and metalloproteinase) family (ADAM-10, -12, and -17) and ADAMTSs (ADAM with thrombospondin motifs) (ADAMTS-1, -4, and -5). TIMP-1 inhibits ADAM-10. While TIMP-1-null mice and TIMP-2-null mice do not exhibit obvious abnormalities, TIMP-3 ablation in mice causes lung emphysema-like alveolar damage [66] and faster apoptosis of mammary epithelial cells after weaning [67], indicating that TIMP-3 is a major regulator of metalloproteinase activities *in vivo*. However, the functions of TIMPs go beyond the inhibition of MMPs and are also partakers in the activation and coactivation of others [68]. The balance between the levels of activated MMPs and free TIMPs determines in part the net MMP activity. In addition to regulating the MMPs, TIMPs have also been shown to have angiogenic and growth factor-like activities [69].

 Numerous studies have demonstrated that specific MMPs and TIMPs are expressed in periprosthetic tissues and are critically involved in the bone resorption and subsequent implant failure (Tables 1 and 2). In a study conducted by Takei and coworkers, the mRNA expression patterns of 16 different types of MMPs in synovium-like interface tissues between bone and prosthesis of loose artificial hip joints were analyzed to evaluate which MMPs were present at the mRNA level and possibly contributed to periprosthetic loosening [70]. It was shown that periprosthetic tissues were characterized by highly elevated expression of MMP-1, MMP-9, MMP-10, MMP-12, and MMP-13; moderate expression of MMP-2, MMP-7, MMP-8, MMP-11, MT1-MMP (MMP-14), MT2-MMP (MMP-15), MT3-MMP (MMP-16), MT4-MMP (MMP-17), and MMP-19; lower expression of MMP-3; and little significance of MMP-20. Quantitative analysis of mRNA expression of their endogenous inhibitors (TIMPs) in periprosthetic tissues showed a significant upregulation of TIMP-1, -2, and -3 mRNA expressions in contrast to the decreased levels of TIMP-4 [71]. On protein level, strong immunoreactivity was observed for the extracellular matrix metalloproteinase inducer (EMMPRIN/CD147) in the lining-like layers, sublining area, and vascular endothelium of synovium-like interface tissue around loosened prostheses. Moreover, double immunofluorescence labeling revealed EMMPRIN/MMP-1 double-positive cells in lining-like areas and the sublining area of interface tissue. These data indicated that EMMPRIN expression was upregulated in interface tissues, and that locally accumulated EMMPRIN may modulate MMP-1 expression [72].

 In another study, Nawrocki and coworkers used immunohistochemistry (IHC) to identify the cells responsible for the synthesis of MMPs in the periprosthetic microenvironment [73]. MMP-2 (gelatinase A) and its activator MT1-MMP were strongly detected in macrophages and multinucleated giant cells in contact with polyethylene wear debris. Similar results have been also obtained by other IHC studies on MMP-2 in this pathological process [58, 74, 75]. Indeed, these studies reported the expression of MMP-2, as well as those of other MMPs, such as MMP-9 and MMP-1 and, in a more restricted pattern, MMP-3, in macrophages, fibroblasts, and endothelial cells. The strong expression of MMP-2 and its activator MT1-MMP in phagocytic cells of periprosthetic samples suggests their contribution to aseptic loosening of prosthetic components. These data are supported by the observation that high levels of gelatinolytic activities were also previously detected in the same type of lesion [76–79]. Of particular interest was the colocalization of MMP-2, MT1-MMP, and TIMP-2 in the same cells [73]. The strong expression of TIMP-2 in interface tissue around implants was also reported by Ishiguro and coworkers [80]. These data support the concept of Strongin and coworkers, who postulated that proMMP-2 activation could be mediated by a trimolecular stoichiometric complex involving MMP-2, TIMP-2, and MT1-MMP [81]. More specifically, these authors demonstrated that the activated form of MT1-MMP acts as a cell surface TIMP-2 receptor. The MT1-MMP/TIMP-2 complex may in turn serve as a receptor for proMMP-2, leading to its processing into the active enzyme. Interestingly, the detection of a soluble type of MT1-MMP (~56 kDa) in synovial and pseudosynovial fluid of patients with rheumatoid arthritis, osteoarthritis, and loose arthroplasty endoprostheses has been previously reported, without clarifying the origin of this type or its activation state. It was proposed that this form was probably processed proteolytically from the transmembrane type of MT1-MMP [75]. A protein band of ~56 kDa was also detected in periprosthetic tissues extracts and pseudosynovial fluids from loose arthroplasty endoprostheses that was ascribed to a soluble form of MT1-MMP [82].

 The contribution of different members of the MMP family in gelatinolytic and collagenolytic potential was evaluated by gelatin zymography, and the degradation of synthetic dinitrophenyl-Pro-Gln-Gly-Ile-Ala-Gly-Gln-D-Arg (DNP-S) together with reverse phase high performance liquid chromatography, respectively [82]. Activated species of both MMP-1 and MMP-13 were identified in most periprosthetic tissues, which could be responsible for the detected DNP-S-degrading activity, while the gelatinases MMP-2 and MMP-9 did not contribute in this potential, since they mainly existed in complex with TIMP-2 and TIMP-1, respectively. These data indicated that MMP-1 and MMP-13 may play a key role in the degradation of periprosthetic ECM, since they degrade native type-I and type-III collagens. Moreover, they may directly contribute to bone resorption, by removing the osteoid layer from calcified bone, facilitating the osteoclastic bone resorption [83–85]. Accordingly, it has been previously reported that periprosthetic tissue extracts exhibited high TIMP-free collagenolytic activity although TIMP-1 and TIMP-2 have been detected in periprosthetic tissues [78, 79]. TIMPs produced by pseudosynoviocytes may be released into synovial fluid to limit MMP proteolysis, but their localization far from local degradation sites leads to the hypothesis of a disruption of the MMP-TIMP balance in favor of MMPs surrounding wear particles.

 Immunohistochemical study of the plasminogen activation system, which is closely associated with MMP activities, disclosed localization in periprosthetic tissues of urokinase plasminogen activator (uPA), uPA-receptor (uPAR), and tissue type plasminogen activator (tPA) in macrophages with phagocytosed metal, polyethylene, cement particles, or accompanying pieces of necrotic bone [86]. Plasminogen activator inhibitor-1 (PAI-1) staining was present in the neighboring areas that stained for uPA or tPA, but PAI-1 staining was also found overlapping and outside these areas. These findings suggest a role for the uPA/uPAR and PAI-1 in activation and focalization of extracellular matrix degradation in periprosthetic tissues. The expression of the plasminogen activation system by macrophages containing phagocytosed material suggests undegradable microdebris as a possible initiating and perpetuating stimulus for a proteolytic activation cascade, which may contribute to loosening of the prosthesis. In contrast to most ECM-degrading proteases, uPA has restricted substrate specificity. Although uPA best-documented proteolytic action is the conversion of inactive plasminogen to active plasmin, it has been also reported that it is able to activate the cell surface MT1-MMP proenzyme [87]. Like uPA, plasmin is also a serine protease but, in contrast to uPA, has broad substrate specificity. Apart from native collagen, plasmin can degrade most proteins present in the ECM. It can also activate the precursor forms of a number of MMPs, such as MMP-3, MMP-9, MMP-12, and MMP-13 [88]. Elevated protein levels of MMP-13, together with uPA and PAI-1 in periprosthetic pseudocapsular and interface tissues were also reported by Diehl and coworkers [89]. However, no significant correlation between the protein expression of these factors and years from arthroplasty to revision or to type of fixation (cemented versus cementless) was observed.

It should be noted that the physical characteristics of wear particles (size, shape, and sintering temperature) as well as their amounts in the periprosthetic tissues can modify the toxicity of the biomaterials and the production of cytokines, MMPs, and TIMPs by various cell types. For example, macrophages seemed to release MMPs (MMP-1, -2, and -9) in proportion to the amount of particulate debris at the prosthetic interface [90]. Laquerriere and coworkers demonstrated that sintering temperature (that modify crystal size and surface area) had little effect on MMPs and TIMPs production. Nonphagocytable particles induced more MMP-9, although phagocytable particles induced more IL-1*β* release. The shape of the particles was the most important factor since needle-shaped particles induced the most significant upregulated expression of MMPs (mostly MMP-9) and IL-1*β* [91]. In another study, human osteoblasts were incubated with particles experimentally generated in the interface between hip stems with rough and smooth surface finishings as well as different material compositions [39]. The results revealed distinct effects on the cytokine release of human osteoblasts towards particulate debris. Thereby, human osteoblasts released increased levels of IL-6 and IL-8 after treatment with metallic wear particles. The expression of VEGF was slightly induced by all particle entities at lower concentrations. Apoptotic rates were enhanced for osteoblasts exposed to all the tested particles. Furthermore, the *de novo *synthesis of type 1 collagen was reduced and the expression of MMP-1 was considerably increased. Therefore, by the secretion of degrading effectors, osteoblasts may actively contribute to matrix weakening.

## 3. Molecular Mechanisms Controlling the Periprosthetic Microenvironment: Implication of MMPs/TIMPs and an Emerging Role for Proteasome

**Figure 2:**
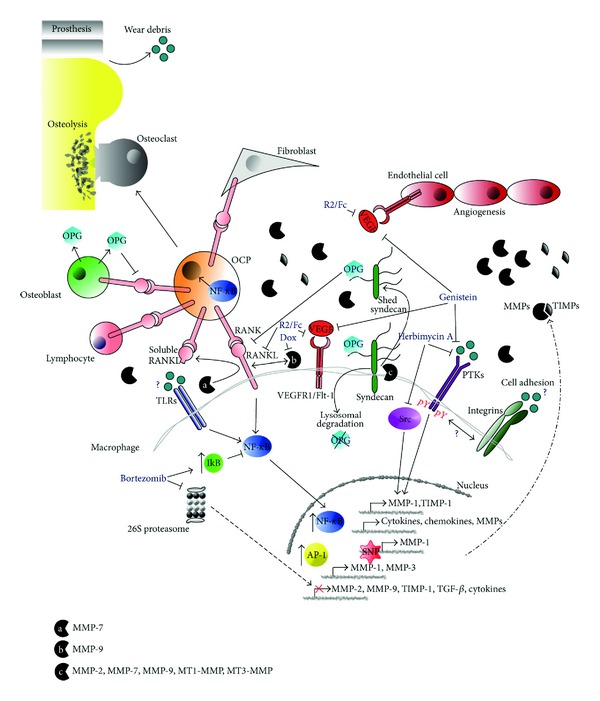
Hypothetical model of the molecular mechanisms that control periprosthetic microenvironment and potential molecular targeting with regard to the expression and activity of MMPs/TIMPs to prevent osteolysis *(see text for details)*.

 A large body of studies reveals a strong interdependence of MMP expression and activity with the molecular mechanisms that control the composition and turnover of periprosthetic ECMs. MMPs can either actively modulate or be modulated by the molecular mechanisms that determine the debris-induced remodeling of the periprosthetic microenvironment (summarized in Figure 2). One likely mechanism whereby particulate debris may induce osteoclast generation and activation is an indirect one, mediated through the actions of proinflammatory mediators that can act on osteoclast precursors and, most importantly, modulate the RANKL/osteoprotegerin (OPG) ratio through actions on cells within the periprosthetic tissue. RANKL is a type II homotrimeric transmembrane protein, which is normally expressed on osteoblastic cell membrane but is also expressed by fibroblasts and activated T cells [92]. Binding of RANKL to RANK on preosteoclasts (OCPs) activates NF-*κ*B and Jun N-terminal kinases (JNKs) pathways to induce cell differentiation [93]. NF-*κ*B is likely the most notable transcription factor implicated in wear debris action. This protein complex, long known as a key regulator of inflammatory gene expression, is also emerging as an important player during osteoclastogenesis. Supporting evidence for a role of NF-*κ*B in periprosthetic osteolysis comes from observations that deficiency of NF-*κ*B in mice protects against titanium-induced calvarial osteolysis [94], and that inhibition of NF-*κ*B blocks wear debris induction of osteoclastogenesis *in vitro* [95, 96]. Osteoblasts also secrete OPG, a soluble decoy receptor for RANKL, which strongly binds to RANKL and effectively inhibits its activity on preosteoclasts differentiation and maturation [97]. OPG is a glycoprotein possessing 4 cysteine-rich domains at its Nterminus by which it binds to RANKL, whereas its C-terminus contains 22 homologous death domains of unknown function and a heparin binding domain by which the glycoprotein interacts with matrix macromolecules, such as glycosaminoglycans and proteoglycans. Importantly, any imbalance in the RANKL/OPG ratio impairs normal bone remodeling and evidence suggests a role of RANKL/OPG ratio in wear debris-induced osteolysis. In particular, it has been shown that RANKL blockade with OPG [98, 99] or RANK:Fc (RANKL antagonist consisting of the extracellular region of RANK fused to the Fc portion of human IgG1), or by using mice genetically deficient in RANK prevents wear debris-induced osteolysis in the murine calvarial model [100]. Moreover, wear debris can increase the RANKL/OPG ratio in murine calvarial tissues [101], and several reports have identified elevated RANKL expression in IFTs [102–105]. However, the fact that several different cell types within the periprosthetic tissue are capable of RANKL expression, including osteoblasts, fibroblasts, T lymphocytes, and also macrophages and giant cells [102–106], makes the cellular basis for elevated RANKL expression very complicated.

 Several MMPs are overexpressed and correlated with osteoclast differentiation, maturation and activation by interfering with the RANK/RANKL/OPG system in inflammation and cancer [107]. MMP-9 is likely to play an important role in the recruitment of osteoclasts at inflammatory and metastatic sites since the use of chemical inhibitors or antisense oligonucleotides against MMP-9 abrogated the recruitment of osteoclasts [108]. Franco and coworkers showed that doxycycline (Dox), which can suppress the enzyme activity of MMP-9 [109], as well as MMP-9 inhibitor (MMP-9 inhibitor I), downregulated the expression of RANKL-induced osteoclast maturation genes in conjunction with the suppression of RANKL-induced osteoclastogenesis [110]. These findings indicated that MMP-9 induced by RANKL plays a role as an upstream effector of osteoclast gene expression, and, as such, it may also be a regulator of osteoclastogenesis. Previous studies reported that MMP inhibitor (RP59794) [111] or MMP-9 gene knockout [112] reduced osteoclast migration, which results in reduction of the resorption process in the growth plate and, as a consequence, attenuated development of bone marrow cavity. However, since the latter studies treated the aspect of osteoclast migration, but not differentiation, the study by Franco and coworkers is the first to report the involvement of MMP-9 activity in RANKL-induced osteoclastogenesis. In another report, MMP-7 could solubilize RANKL in mouse models of prostate and breast cancer promoting osteoclast activation and osteolysis [113]. The limiting step in RANKL-dependent osteoclastogenesis is the contact of RANKL-expressing osteoblasts with RANK on the cell surface of osteoclasts. This limitation is prohibited by proteolytic cleavage of RANKL from the cell surface through the action of MMP-7 and cathepsin G. Importantly, it has been shown in tumor-induced bone disease that soluble RANKL retains its activity and is liberated at the tumor-bone interface promoting osteoclastogenesis without the necessity of direct interaction of osteoblast with osteoclasts [113–115].

Possible accumulation of cell membrane and matrix proteoglycans at the inflammatory periprosthetic ECM may also modulate the RANK/RANKL/OPG system through both MMP-independent and -dependent manners. For example, it has been shown that myeloma cells decrease OPG availability by internalizing it through binding to glycosaminoglycan side chains of surface syndecan-1 and degradation to lysosomes, thereby regulating its inhibitory effect on RANKL [116]. Moreover, shed syndecan-1 secreted by myeloma cells may also bind OPG [117] and block its inhibitory activity to RANKL triggering further osteoclast differentiation and activation. Syndecan ectodomain shedding is an important regulatory mechanism, because it rapidly changes cell surface receptor dynamics and generates soluble ectodomains that can function as paracrine or autocrine effectors or competitive inhibitors. Strong evidence indicates the involvement of several MMPs in syndecan cleavage *in vitro* and *in vivo* [118]. Matrilysin (MMP-7) cleaves syndecan-1 [119], gelatinases MMP-2 and MMP-9 can cleave syndecans-1, -2, and -4 [120, 121], whereas the membrane-associated metalloproteinases MT1-MMP and MT3-MMP are known to cleave syndecan-1 [122]. Taken together, these data suggest a critical role of certain members of MMPs in interfering with the RANK/RANKL signaling axis by directly and/or indirectly regulating OPG and RANKL availability, thereby modulating osteoclast generation and activation within the periprosthetic tissue (Figure 2).

 An important observation in several studies was that specific gene responses were induced in different cell types of the periprosthetic microenvironment by an initial and early particulate biomaterial-cell interaction. The differential gene expression indicated that particle-cell interactions activated specific signaling events and transcription factors. Vermes and coworkers have found that particles rapidly activated protein tyrosine phosphorylation and induced the nuclear transcription factor NF-*κ*B in osteoblasts [123]. The rapid kinetics of the activation suggested that the particles elicited signals before the phagocytosis process. Importantly, inhibition of NF-*κ*B function by either tyrosine kinase inhibitors or antioxidants reversed the suppressive effect of titanium particles on procollagen a1[I] gene expression suggesting a functional relationship in osteoblasts between tyrosine phosphorylation, NF-*κ*B activation, and collagen gene expression. Thus, particle-cell interactions before their phagocytosis appear to initiate an intracellular tyrosine phosphorylation cascade that targets the nuclear activation of the inducible transcription factor NF-*κ*B.

 A role for protein tyrosine kinases (PTKs) in regulation of the activation of MMPs/TIMPs in ion-induced activation of macrophages was suggested by Luo and coworkers [124]. In particular, cobalt (Co) and chromium (Cr) ions, two corrosion products found in the periprosthetic environment of metal-on-metal prostheses, were shown to upregulate MMP-1, TIMP-1, and cytokines (such as TNF-*α*) in cultures of human U937 macrophages. The inhibitory effect of genistein suggested the implication of PTKs in the induction of MMP-1 and TIMP-1 expressions by Co^2+^ and Cr^3+^ ions in macrophages, the most important cellular target of wear debris. Genistein, a soy isoflavonoid that is a natural broad spectrum PTK (such as EGFR, PDGFR, IGFR, Src) inhibitor, has been shown to regulate the transcription of several MMPs and their endogenous inhibitors (TIMPs) by breast cancer cells [125, 126]. Moreover, previous *in vitro* studies demonstrated that genistein downregulates the expression of vascular endothelial growth factor (VEGF), which is a major signaling protein that contributes to angiogenesis [127]. VEGF is produced by multiple cell types, including macrophages and osteoblasts [128, 129]. It exerts its biological activity by binding to two TK receptors, VEGF receptor-1 (VEGFR-1; Flt-1) and VEGFR-2 (Flk-1/KDR) [130]. VEGF is actively involved in the process of inflammation, osteoclastogenesis, and bone resorption [131–133] and probably plays an important role in wear debris-induced inflammatory osteolysis since the periprosthetic tissues at the bone-implant interface show a high degree of vascularization [134]. Notably, Luo and coworkers showed a more potent effect of herbimycin A, Src kinase-specific inhibitor, on the expression of MMP-1 and TIMP-1 compared to genistein [124], providing strong evidence for a critical role of Src kinases in modulating the expression levels of MMP-1 and TIMP-1 in macrophages in the presence of Co^2+^ and Cr^3+^ ions (Figure 2). Other ions released from hip prostheses, such as titanium [135] and nickel [136], have been shown to stimulate TNF-a in a manner similar to Co and Cr, suggesting that other ions may also modulate tyrosine kinase activity probably affecting the amounts and activities of MMPs/TIMPs in periprosthetic ECMs.

 Moreover, the adhesion of macrophages to phosphorylcholine-polymer coated surfaces stimulated the expression of MMP-1 and TIMP-1, suggesting that cell adhesion induced a remodeling of the macrophage ECM. The inhibition of the expression of these genes by genistein and herbimycin A suggested that PTKs were also implicated in this remodeling [124]. Interestingly, this kind of materials-stimulated expression of genes implicated in ECM remodeling was also observed in fibroblasts induced by three-dimensional collagen [137, 138]. In these studies, alpha1beta1 and alpha2beta1 integrins mediated the signals inducing downregulation of collagen gene expression and upregulation of MMP-1, respectively. Therefore, the potential impact of macrophage surface integrins-evoked signals on the periprosthetic microenvironment should be further investigated to better understand the cellular effects of particles liberated from the articular surface of prostheses (Figure 2).

 One major part of the organism's first line of defense against infection is a family of pattern recognition receptors (PRRs) called the Toll-like receptors (TLRs). TLRs are transmembrane proteins found in various cells and recognize infectious and endogenous threats, so-called danger signals, which evoke inflammation and assist adaptive immune reactions. It has been suggested that TLRs play a role in periprosthetic tissues and arthritic synovium. Tamaki and coworkers found that peri-implant tissues were well equipped with TLRs and, in aseptic loosening, monocytes/macrophages were the main TLR-expressing cells [139]. This could lead to production of inflammatory cytokines and MMPs after phagocytosis of wear debris derived from an implant. A major conclusion of the study was that inflammatory cells in both aseptic and septic tissues were equipped with TLRs, providing them with responsiveness to both endogenous and exogenous TLR ligands. In this line, the high expression of TLRs in the periprosthetic tissues could be potentially important, as they can reflect occurrence of subclinical biofilms on the prosthetic surfaces. Activation of TLRs has been suggested to modulate the expression levels of certain MMPs but not TIMPs. In a recent study, Lisboa and coworkers showed that activation of TLR-2 and TLR-4, two TLR members expressed by a variety of human cells that participate in the recognition of bacterial lipoproteins and lipopolysaccharides (LPS) [140], induced an increase in the secretion of MMPs-1, -3, and -10 by cultured periodontal fibroblasts, and this was mediated via the p38, JNK1/2, and NF-*κ*B pathways [141]. It is likely that there is a broad variation in the response of cells to TLR ligands that is dependent on the type of stimulus in the periprosthetic microenvironment. Therefore, the possibility of potentiation of MMPs activation concomitant with TLR activation in periprosthetic tissues needs to be further investigated (Figure 2).

 Genetic variation may determine individual responses in terms of susceptibility to osteolysis and recovery. Expression levels of the MMPs at both the mRNA and protein levels can be affected by the introduction or loss of transcription binding sites by single nucleotide polymorphisms (SNPs). SNPs are the most common sequence variation in the human genome and can affect coding sequences, splicing, or transcription regulation. In a case control, it was shown that a single-nucleotide polymorphism (SNP) of MMP-1 was highly associated with total hip replacement aseptic failure [142]. This SNP existed within a promoter region of the gene and as such may have a direct effect on the amount of gene expression (Figure 2). However, the mechanisms of MMP gene regulation are still not fully delineated, and it is likely that many more functionally important elements in their promoter regions are yet to be identified [143–145]. Moreover, investigation of SNPs in the TIMP genes would be a necessary complement for any study of MMP SNPs, given the evidence that the MMP-to-TIMP ratio plays a role in defining overall MMP activity.

 In another line of research, Ortiz-Lazareno and coworkers found that the proteasome inhibitor MG-132 significantly diminished proinflammatory cytokines (TNF-*α*, IL-1*β*, IL-6) release by U937 macrophages, whereas, induced a decrease in the membrane receptors TNF-R1 and IL-1R1 and an increase in the soluble receptors sTNF-R1 and sIL-1R1. However, MG-132 increased the IL-6R and decreased sIL-6R [146]. In another report, Mao and coworkers investigated the effects of Ti particles and the specific proteasome inhibitor bortezomib on the secretory profile of inflammatory cytokines, chemokines, and inflammatory enzymes in a murine macrophage cell line [147]. It was shown that Ti particles increased the production of TNF-*α*, IL-1*β*, IL-6, IL-10, MCP-1, MIP-1*α*, iNOS, and COX-2 in this cell line, while bortezomib inhibited the expression of all factors, except IL-10, in a time-dependent manner. Bortezomib, a potent, reversible and selective inhibitor of the chymotryptic activity of the proteasome, prevents the degradation of I*κ*B proteins, which mask the nuclear localization sequence of NF-*κ*B, therefore inhibiting the translocation of NF-*κ*B into the nucleus and further inhibiting the transcription and secretion of inflammatory mediators. It is known that NF-*κ*B regulates the transcription of a variety of genes of inflammatory cytokines (TNF-*α*, IL-1*β*, IL-2, IL-6, and GM-CSF (granulocytemacrophage colony-stimulating factor)), chemokines (IL-8, MCP-1, and MIP-1*α*), and inflammatory enzymes (iNOS and COX-2) [148]. Therefore, bortezomib may inhibit the proinflammatory factors mentioned earlier through inhibition of NF-*κ*B activity. Moreover, proteasome inhibition with bortezomib alters the binding of other transcription factors to the promoter region of several molecular effectors, thus modulating their expression levels [149]. The anti-inflammatory cytokine IL-10 induced by bortezomib inhibits TNF-*α* gene expression via inhibiting NF-*κ*B activity or by directly inhibiting TNF-*α* itself [150, 151]. It should be noted that bortezomib (as well as other proteasome inhibitors) exhibits beneficial effect on bone metabolism as it inhibits osteoclastic function and promotes osteoblastic activity by inhibiting NF-*κ*B activation induced by the RANK-RANKL signaling axis, which is the master regulator of differentiation and activation of osteoclasts [152–155].

 The inhibitory effects of bortezomib and MG-132 on the secretion of inflammatory cytokines and their receptors by macrophages suggested the potential involvement of the proteasome pathway in periprosthetic loosening and osteolysis process. The proteasome is a major cellular protease complex that functions as the main driver of intracellular degradation of a wide variety of cellular proteins implicated in several physiological and pathological cellular functions [156]. Interestingly, the proteasome pathway controls via transcriptional and posttranslational mechanisms the concentration and turnover of several ECM macromolecules (including proteoglycans/glycosaminoglycans, MMPs/TIMPs, and collagens) [157]. Importantly, the proteasome provides a link between the regulation of extracellular proteolytic events to intracellular proteolysis by modulating MMP/TIMP expression and activity. In particular, it has been shown that proteasome blockade by proteasome inhibitors resulted in a marked modification of gene expression and activity of MMPs (upregulation of MMP-1, -3 and downregulation of MMP-2, -9) and TIMPs (downregulation of TIMP-1). Moreover, proteasome inhibition regulated also the synthesis and activity of other ECM constituents, such as TGF-*β* (downregulation), decorin (upregulation), and collagen type-I and type-IV (downregulation) [157]. Therefore, since matrix remodeling and degradation can be tightly regulated by proteasome activities, its modulation may be considered as a novel strategy to control the properties of periprosthetic ECMs as has been recently suggested for tumor microenvironment (Figure 2).

## 4. Potential Therapeutic Perspectives

 Much progress has recently been made in understanding the molecular and cellular mechanisms whereby prosthetic wear debris can ultimately cause aseptic loosening and osteolysis. However, the complex nature of the interactions between wear particles and periprosthetic cells as well as the multiple intracellular signaling pathways activated by such interactions results in the reality that development of therapeutic approaches to the treatment of periprosthetic osteolysis is long overdue. In the following lines, we will try to address strong rationale for potential clinical applications of the described molecular mechanisms for periprosthetic loosening and osteolysis treatment. 

 The finding that proteasome inhibitors (i.e., bortezomib, MG-132) altered the macrophage secretory profile of inflammatory cytokines, chemokines, and inflammatory enzymes [146, 147], which play a key-role in the inflammatory response of periprosthetic tissue to wear debris, reveals a critical role for proteasome in the development of periprosthetic loosening and osteolysis. In a recent review, we have highlighted the novel approach of targeting the proteasome as a mechanism to control the synthesis and bioactivity of ECM effectors in tumors, since the proteasome appears to be an elegant molecular regulator of specific matrix macromolecules [157]. From the data described in the present review, proteasome signaling pathway emerges as a promising target to selectively regulate the synthesis and activity of inflammatory factors (such as TNF-*α*, IL-1*β*, IL-6, IL-8), their membrane receptors, and matrix degrading effectors (such as specific MMPs) in the periprosthetic microenvironment. To this aim, a promising agent is bortezomib, which exhibits multiple functions by interfering also with other intracellular signaling pathways such as the RANK-RANKL system thereby regulating new bone formation by both inducing osteoblastic function and inhibiting osteoclastogenesis. However, the elevated production of reactive oxygen species (ROS) by activated macrophages and osteoclasts in the presence of wear particles [158] should be considered in this context, since proteasome inhibitors have been also shown to induce ROS [159], which further attenuate the proteasomal system activation [160]. This proteasomal inhibition would potentially result in the accumulation of phosphorylated c-Jun and activation of AP-1 that ultimately induce MMP-1 and MMP-3 expression levels [160]. Therefore, proteasome inhibitors may have a synergistic effect with wear particles on ROS production and strongly induce the expression of specific MMPs within the periprosthetic microenvironment although their inhibitory effect on inflammatory cytokines and their receptors as well as other MMPs has been documented. Moreover, MG-132 has been found to significantly downregulate TIMP-1 expression in organ interface tissue cultures and primary IFT fibroblast cultures (Aletras and coworkers, unpublished data), which is in line with the findings of Fineschi and coworkers in dermal fibroblasts [161], revealing the complex and questionable role of proteasome in regulating distinct molecular effectors that would potentially be beneficial for periprosthetic osteolysis treatment. Therefore, the efficacy of proteasome inhibitors (such as bortezomib) to prevent periprosthetic loosening and osteolysis caused by implant-derived particles is an emerging concept and needs to be further investigated.

 Taking under consideration these data, it should be investigated whether an alternative strategy associated with proteasome activation would be more beneficial in the treatment of periprosthetic loosening. Several activators of the proteasome, such as isoflavonoids, should be tested in order to reverse the effects on the expression levels of specific MMPs and TIMPs described previously, as a result of the reduced proteasome activity in IFT. Proteasome activation might be further induced by combined treatment with activators of nuclear factor erythroid 2-related factor 2 (Nrf2), such as sulforaphane [162]. Notably, Nrf2 upregulates the transcription of multiple antioxidant enzymes providing an effective means of reducing elevated ROS levels in IFT.

 The observation that genistein and herbimycin A strongly attenuated the expression of MMP-1 and TIMP-1 by macrophages implied that tyrosine kinases play also an essential role in the signaling pathways regulating the remodeling of macrophage ECM in the periprosthetic microenvironment [124]. Therefore, PTKs (e.g., Src kinases) may serve as an additional target for selective inhibition of periprosthetic osteolysis. Importantly, proteasome is implicated in this process since it has been reported that herbimycin A targets the degradation of tyrosine kinases by the 20S proteasome [163]. Moreover, apart from its inhibitory action on PTKs, genistein was found to downregulate the expression of VEGF, a major angiogenic factor in periprosthetic microenvironment. The interactive network of the VEGF/Flt-1 and RANKL/RANK pathways may play important roles in the initiation, progression, and resolution of aseptic loosening. In a study by Ren and coworkers, it was shown that VEGF may be actively involved in the regulation of RANK/RANKL gene expression, and that it exerted a regulatory effect on the development of particle-induced inflammatory osteoclastogenesis through its unique Flt-1, rather than Flk-1, receptor located on monocyte/macrophage cell lineages [164]. In particular, they found that treatment with R2/Fc (a VEGF neutralizing antibody) but not SU5416 (an Flk-1 receptor inhibitor) resulted in the inhibition of polyethylene particle-enhanced VEGF/Flt-1 signaling and inflammatory osteolysis by trapping VEGF in the periprosthetic milieu (Figure 2). Taken together, these findings provide the biological rationale for a combined VEGF/Flt-1- and RANKL/RANK-targeted treatment strategy, especially in the early stages of wear debris-induced inflammatory response. The fact that the RANK/RANKL/OPG system is of crucial importance for the development of periprosthetic osteolysis together with the finding that Dox inhibits RANKL-induced osteoclastogenesis by its inhibitory effect on MMP-9 enzyme activity [110] provides a reasonable rationale for a pharmaceutical advantage of tetracycline antibiotics against periprosthetic osteolysis. It should be noted that this class of antibiotics, including Dox, has been effectively utilized for the treatment of bone resorptive diseases because of their activity to suppress osteoclastogenesis induced by RANKL.

 Given that excessive osteoclast activity represents the cellular endpoint of osteolysis, it is not surprising that the bisphosphonate class of osteoclast inhibitors have come in for much discussion as possible therapeutic agents for this disease. Again, however, despite promising results in animal models, there is no clinical evidence supporting the effectiveness of these drugs in the treatment of osteolysis patients. Alendronate inhibits wear debris-induced osteolysis in the rat loaded tibial implant model of osteolysis [165] and in a similar canine model [9] and is also effective in preventing osteolysis in the murine calvarial model [6]. A single dose of zoledronic acid administered directly after surgery also suppressed particle-induced osteolysis in mouse calvaria [166]. Bisphosphonates inhibit osteoclast formation by blocking the mevalonate pathway of isoprenoid biosynthesis. Their potential effect in periprosthetic osteolysis should be also considered with regard to their ability to inhibit the enzymatic activity of various MMPs. Certain bisphosphonates showed beneficial effects as a result of altering the expression pattern of MMPs/TIMPs by inhibiting and increasing the gene and protein expression of several MMPs and TIMPs, respectively, in breast cancer cells. In particular, it has been shown that zoledronic acid suppressed the expression of metalloproteinases MMP-2, -9, the membrane type MT1- and MT2-MMP, whereas it increased the expression of their endogenous tissue inhibitors [167].

 Though not extensively studied, other mechanisms that should be further investigated with regard to their contribution to the remodeling of periprosthetic ECM include SNPs of certain MMP/TIMP genes as well as the involvement of TLRs in periprosthetic inflammation. An SNP of MMP1 gene was highly associated with total hip replacement aseptic failure [142]. It should be noted that MMPs do not possess only degrading functions but they also play protective and anti-inflammatory roles. Therefore, the association that exists with a particular polymorphic form of MMP-1 does not necessarily show that particular form is associated with increased MMP-1 activity; in fact, the opposite may be true. The possibility that SNP markers may serve as predictors of implant survival and aid in pharmacogenomic prevention of total joint replacement failure should be further investigated. A more comprehensive analysis of MMP and TIMP SNPs is thus required, and given the coverage by existing genome-wide association study (GWAS) platforms, a candidate gene approach is justified. Regarding TLRs, strong evidence indicated that macrophages, which are the most important cellular targets of wear debris, are the main TLR-expressing cells in periprosthetic microenvironment. The increased secretion of MMPs by combined TLR activation may be an important factor that should also be considered during treatment of periprosthetic loosening and osteolysis.

 Extended information is available regarding the action of several nonsteroidal anti-inflammatory drugs (NSAIDs) upon significant for the loosening process effector molecules, which though originates from *in vitro* studies with articular chondrocytes and synovial or dental pulp fibroblasts [168–173]. However, little information is available in the literature about their possible role in retarding the periprosthetic loosening and bone resorption process. To this aim, we tested the effect of four widely used NSAIDS (i.e., aceclofenac, piroxicam, tenoxicam, and indomethacin) on cytokine, MMP, TIMP, and prostanoid production by IFT from patients with aseptic loosening of total arthroplasty [174]. The results showed that all the tested drugs exerted uniformly an inhibitory effect on IL-6 and TNF-*α*, both known to directly cause osteoclastic bone resorption, independently of PGE2 [175–177]. Moreover, all of them modified specific MMPs (MMP-1, MMP-2, MMP-3, and MMP-9) expression and activity, although these drugs did not have a statistically clear effect on MMPs, which might reflect individual responses in terms of susceptibility to osteolysis. However, NSAIDs had a profound stimulatory effect on TIMP-1 production. Interestingly, paracetamol, which was used as a neutral drug, significantly decreased the synthesis of TNF-*α* and gelatinases (MMP-2 and MMP-9). Considering these observations, NSAIDs could reduce the ability of periprosthetic membrane to cause bone resorption, which is in line with previous reports that have shown that piroxicam, which exhibited about the same effects as the other tested NSAIDs, significantly decreased the IFT-induced resorptive process [178]. Consequently, *in vivo* long-term clinical trials may shed light on the possibility of a beneficial effect of specific NSAIDs on the loosening process.

## 5. Concluding Remarks

 The elucidation and understanding of the cellular and molecular mechanisms that control the composition, turnover, and activity of matrix macromolecules within the periprosthetic microenvironment exposed to wear debris is highly important for the development of novel therapeutic approaches to the treatment of periprosthetic loosening and osteolysis. One ultimate target would be to disrupt the vicious cycle between the inflammatory response to wear debris particles induced by the secreted proinflammatory and osteoclastogenic cytokines and the periprosthetic osteolytic cascade governed by the uncontrolled action of MMPs. Considering the multicomplex biological mechanisms underlying the particle-induced periprosthetic loosening and osteolysis described in the present review, it may be crucial to develop and use combinations of conventional therapeutic agents as well as new approaches targeting specific extracellular, cell surface, and intracellular molecular effectors and apply them in clinical practice. 

## Figures and Tables

**Table 1:** Matrix metalloproteinases (MMPs) in periprosthetic microenvironment (expression and/or activity: ↑ with bold data: high; ↑ without bold data: moderate).

MMPs		Substrates	Expression and/or activity in periprosthetic microenvironment [References]
*Collagenases *			
Contain hemopexin domain and peptide linking with catalytic domain	**MMP-1** (interstitial collagenase; collagenase 1) **MMP-8** (neutrophil collagenase; collagenase 2) **MMP-13** (collagenase 3)	Collagen type I, III, V, VII, VIII, X, gelatin, IL-1*β*, MMP-2, -9, fibronectin	**↑MMP-1** [39, 58, 70, 72, 80, 82, 90] ↑MMP-8 [70] **↑MMP-13** [70, 82, 89]

*Gelatinases *			
High substrate specificity to native collagen and gelatin	**MMP-2** (gelatinase A; 72 kDa metalloproteinase) **MMP-9** (gelatinase B; 92 kDa metalloproteinase)	Collagen type IV, V, VII, X, proteoglycans, gelatin, elastin, laminin	**↑MMP-2** [58, 70, 73, 74, 76, 77, 80, 90] **↑MMP-9** [58, 70, 76, 77, 80, 90, 91]

*Stromelysins *			
Metalloproteinases of stroma	**MMP-3** (stromelysin 1) **MMP-10** (stromelysin 2) **MMP-11** (stromelysin 3)	Proteoglycans, fibronectin, laminin, elastin, gelatin, plasminogen, vitronectin, fibrinogen, fibrin, collagen type III, IV, V, antithrombin III, MMP-1, -2, -8, -9, -13	↑MMP-3 [58, 70, 74, 80] **↑MMP-10** [70] ↑MMP-11 [70]

*Matrilysins *			
The smallest among MMPs, lack of hemopexin domain	**MMP-7** (matrilysin, metalloendopeptidase) **MMP-26** (matrilysin-2, endometase)	Collagen type IV, proteoglycans, glycoproteins, gelatin	↑MMP-7 [70]

*Membrane-type MMPs *			
(A) Transmembrane-type MMPs	**MMP-14** (MT1-MMP) **MMP-15** (MT2-MMP) **MMP-16** (MT3-MMP) **MMP-24** (MT5-MMP)	Collagen type I, II, III, gelatin, elastin, laminin, fibronectin, fibrin, proteoglycans, proMMP-2, proMMP-13	**↑MMP-14** [70, 73] ↑MMP-15 [70] ↑MMP-16 [70]
(B) GPI-anchored MMPs	**MMP-17** (MT4-MMP) **MMP-25** (MT6-MMP)		↑MMP-17 [70]

*Other MMPs *			
MMPs that are not categorized in any of the previous groups	**MMP-12** (macrophage metalloelastase) **MMP-19** **MMP-20** (enamelysin) **MMP-21, MMP-23** **MMP-27, MMP-28**		**↑MMP-12** [70] ↑MMP-19 [70]

**Table 2:** Tissue inhibitors of metalloproteinases (TIMPs) in periprosthetic microenvironment (expression and/or activity: ↑ with bold data: high; ↓ without bold data: low).

TIMPs	Preferred MMP/ADAM/ADAMTS	Expression and/or activity in periprosthetic microenvironment [References]
TIMP-1	Most MMPs, ADAM-10 (inhibition). MT3-MMP, MT5-MMP, MMP-19 (weak inhibition)	**↑TIMP-1** [71, 74, 76, 78, 80, 82]
TIMP-2	Most MMPs (inhibition). MMP-2 (activation)	**↑TIMP-2** [71, 73, 76, 80, 82]
TIMP-3	Most MMPs, ADAM-10, -12, -17, and ADAMTS-1, -4, -5 (inhibition). MMP-2, MT3-MMP (activation)	**↑TIMP-3** [71]
TIMP-4	Most MMPs (inhibition)	↓TIMP-4 [71]

## References

[1] Sundfeldt M, Carlsson LV, Johansson CB, Thomsen P, Gretzer C (2006). Aseptic loosening, not only a question of wear: a review of different theories. *Acta Orthopaedica*.

[2] Dumbleton JH, Manley MT, Edidin AA (2002). A literature review of the association between wear rate and osteolysis in total hip arthroplasty. *Journal of Arthroplasty*.

[3] Schmalzried TP, Jasty M, Harris WH (1992). Periprosthetic bone loss in total hip arthroplasty. Polyethylene wear debris and the concept of the effective joint space. *Journal of Bone and Joint Surgery A*.

[4] Hirakawa K, Bauer TW, Stulberg BN (1996). Comparison and quantitation of wear debris of failed total hip and total knee arthroplasty. *Journal of Biomedical Materials Research*.

[5] Margevicius KJ, Bauer TW, McMahon JT, Brown SA, Merritt K (1994). Isolation and characterization of debris in membranes around total joint prostheses. *Journal of Bone and Joint Surgery A*.

[6] Schwarz EM, Benz EB, Lu AP (2000). Quantitative small-animal surrogate to evaluate drug efficacy in preventing wear debris-induced osteolysis. *Journal of Orthopaedic Research*.

[7] Millett PJ, Allen MJ, Bostrom MPG (2002). Effects of alendronate on particle-induced osteolysis in a rat model. *Journal of Bone and Joint Surgery A*.

[8] Merkel KD, Erdmann JM, McHugh KP, Abu-Amer Y, Ross FP, Teitelbaum SL (1999). Tumor necrosis factor-*α* mediates orthopedic implant osteolysis. *American Journal of Pathology*.

[9] Shanbhag AS, Hasselman CT, Rubash HE (1997). The John Charnley Award. Inhibition of wear debris mediated osteolysis in a canine total hip arthroplasty model. *Clinical Orthopaedics and Related Research*.

[10] Wooley PH, Morren R, Andary J (2002). Inflammatory responses to orthopaedic biomaterials in the murine air pouch. *Biomaterials*.

[11] Warme BA, Epstein NJ, Trindade MCD (2004). Proinflammatory mediator expression in a novel murine model of titanium-particle-induced intramedullary inflammation. *Journal of Biomedical Materials Research B*.

[12] Yang S-Y, Wu B, Mayton L (2004). Protective effects of IL-1Ra or vIL-10 gene transfer on a murine model of wear debris-induced osteolysis. *Gene Therapy*.

[13] Blaine TA, Rosier RN, Puzas JE (1996). Increased levels of tumor necrosis factor-*α* and interleukin-6 protein and messenger RNA in human peripheral blood monocytes due to titanium particles. *Journal of Bone and Joint Surgery A*.

[14] Nakashima Y, Sun D-H, Trindade MCD (1999). Signaling pathways for tumor necrosis factor-*α* and interleukin-6 expression in human macrophages exposed to titanium-alloy particulate debris *in vitro*. *Journal of Bone and Joint Surgery A*.

[15] Wimhurst JA, Brooks RA, Rushton N (2001). Inflammatory responses of human primary macrophages to particulate bone cements *in vitro*. *Journal of Bone and Joint Surgery B*.

[16] Ingham E, Green TR, Stone MH, Kowalski R, Watkins N, Fisher J (2000). Production of TNF-*α* and bone resorbing activity by macrophages in response to different types of bone cement particles. *Biomaterials*.

[17] Maloney WJ, James RE, Smith RL (1996). Human macrophage response to retrieved titanium alloy particles *in vitro*. *Clinical Orthopaedics and Related Research*.

[18] Saleh KJ, Thongtrangan I, Schwarz EM (2004). Osteolysis: medical and surgical approaches. *Clinical Orthopaedics and Related Research*.

[19] Purdue PE, Koulouvaris P, Nestor BJ, Sculco TP (2006). The central role of wear debris in periprosthetic osteolysis. *HSS Journal*.

[20] Schmalzried TP, Jasty M, Harris WH (1992). Periprosthetic bone loss in total hip arthroplasty. Polyethylene wear debris and the concept of the effective joint space. *Journal of Bone and Joint Surgery A*.

[21] Glant TT, Jacobs JJ, Molnar G, Shanbhag AS, Valyon M, Galante JO (1993). Bone resorption activity of particulate-stimulated macrophages. *Journal of Bone and Mineral Research*.

[22] Goppelt-Struebe M, Stroebel M (1995). Synergistic induction of monocyte chemoattractant protein-1 (MCP-1) by platelet-derived growth factor and interleukin-1. *FEBS Letters*.

[23] Horowitz SM, Gonzales JB (1996). Inflammatory response to implant particulates in a macrophage/osteoblast coculture model. *Calcified Tissue International*.

[24] Ishiguro N, Kojima T, Ito T (1997). Macrophage activation and migration in interface tissue around loosening total hip arthro­plasty components. *Journal of Biomedical Materials Research*.

[25] Kadoya Y, Revell PA, Al-Saffar N, Kobayashi A, Scott G, Freeman MAR (1996). Bone formation and bone resorption in failed total joint arthroplasties: histomorphometric analysis with histochemical and immunohistochemical technique. *Journal of Orthopaedic Research*.

[26] Pizzoferrato A, Stea S, Sudanese A (1993). Morphometric and microanalytical analyses of alumina wear particles in hip prostheses. *Biomaterials*.

[27] DeHeer DH, Engels JA, DeVries AS (2001). In situ complement activation by polyethylene wear debris. *Journal of Biomedical Materials Research*.

[28] Santavirta S, Hoikka V, Eskola A, Konttinen YT, Paavilainen T, Tallroth K (1990). Aggressive granulomatous lesions in cementless total hip arthroplasty. *Journal of Bone and Joint Surgery B*.

[29] Rezzonico R, Chicheportiche R, Imbert V, Dayer J-M (2000). Engagement of CD11b and CD11c *β*2 integrin by antibodies or soluble CD23 induces IL-1*β* production on primary human monocytes through mitogen- activated protein kinase-dependent pathways. *Blood*.

[30] Rezzonico R, Imbert V, Chicheportiche R, Dayer J-M (2001). Ligation of CD11b and CD11c *β*2 integrins by antibodies or soluble CD23 induces macrophage inflammatory protein 1*α* (MIP-1*α*) and MIP-1*β* production in primary human monocytes through a pathway dependent on nuclear factor-*κ*B. *Blood*.

[31] Palecanda A, Paulauskis J, Al-Mutairi E (1999). Role of the scavenger receptor MARCO in alveolar macrophage binding of unopsonized environmental particles. *The Journal of Experimental Medicine*.

[32] Rakshit DS, Lim JTE, Ly K (2006). Involvement of complement receptor 3 (CR3) and scavenger receptor in macrophage responses to wear debris. *Journal of Orthopaedic Research*.

[33] Sun D-H, Trindade MCD, Nakashima Y (2003). Human serum opsonization of orthopedic biomaterial particles: protein-binding and monocyte/macrophage activation *in vitro*. *Journal of Biomedical Materials Research A*.

[34] Horiki M, Nakase T, Myoui A (2004). Localization of RANKL in osteolytic tissue around a loosened joint prosthesis. *Journal of Bone and Mineral Metabolism*.

[35] Quinn JMW, Horwood NJ, Elliott J, Gillespie MT, Martin TJ (2000). Fibroblastic stromal cells express receptor activator of NF-*κ*B ligand and support osteoclast differentiation. *Journal of Bone and Mineral Research*.

[36] Yao J, Glant TT, Lark MW (1995). The potential role of fibroblasts in periprosthetic osteolysis: fibroblast response to titanium particles. *Journal of Bone and Mineral Research*.

[37] Manlapaz M, Maloney WJ, Smith RL (1996). *In vitro* activation of human fibroblasts by retrieved titanium alloy wear debris. *Journal of Orthopaedic Research*.

[38] Vermes C, Chandrasekaran R, Jacobs JJ, Galante JO, Roebuck KA, Glant TT (2001). The effects of particulate wear debris, cytokines, and growth factors on the functions of MG-63 osteoblasts. *Journal of Bone and Joint Surgery A*.

[39] Lochner K, Fritsche A, Jonitz A (2011). The potential role of human osteoblasts for periprosthetic osteolysis following exposure to wear particles. *International Journal of Molecular Medicine*.

[40] Sabokbar A, Kudo O, Athanasou NA (2003). Two distinct cellular mechanisms of osteoclast formation and bone resorption in periprosthetic osteolysis. *Journal of Orthopaedic Research*.

[41] Wang C-T, Lin Y-T, Chiang B-L, Lee S-S, Hou S-M (2010). Over-expression of receptor activator of nuclear factor-*κ*B ligand (RANKL), inflammatory cytokines, and chemokines in periprosthetic osteolysis of loosened total hip arthroplasty. *Biomaterials*.

[42] Möller B, Villiger PM (2006). Inhibition of IL-1, IL-6, and TNF-*α* in immune-mediated inflammatory diseases. *Springer Seminars in Immunopathology*.

[43] Horai R, Saijo S, Tanioka H (2000). Development of chronic inflammatory arthropathy resembling rheumatoid arthritis in interleukin I receptor antagonist-deficient mice. *The Journal of Experimental Medicine*.

[44] Caicedo M, Jacobs JJ, Hallab NJ (2010). Inflammatory bone loss in joint replacements: the mechanisms. *Journal Musculoskeletal Medicine*.

[45] Lerner UH, Ohlin A (1993). Tumor necrosis factors *α* and *β* can stimulate bone resorption in cultured mouse calvariae by a prostaglandin-independent mechanism. *Journal of Bone and Mineral Research*.

[46] Horowitz SM, Purdon MA (1995). Mediator interactions in macrophage/particulate bone resorption. *Journal of Biomedical Materials Research*.

[47] Chiba J, Rubash HE, Kim KJ, Iwaki Y (1994). The characterization of cytokines in the interface tissue obtained from failed cementless total hip arthroplasty with and without femoral osteolysis. *Clinical Orthopaedics and Related Research*.

[48] Takei H, Pioletti D, Kwon SY, Sung P (2000). Combined effect of titanium particles and TNF-a on the production of IL-6 by osteoblast-like cells. *Journal of Biomedical Materials Research*.

[49] Gowen M, Wood DD, Ihrie EJ, McGuire MK, Russell RG (1983). An interleukin 1 like factor stimulates bone resorption *in vitro*. *Nature*.

[50] Akatsu T, Takahashi N, Udagawa N (1991). Role of prostaglandins in interleukin-1-induced bone resorption in mice *in vitro*. *Journal of Bone and Mineral Research*.

[51] Goodman SB, Chin RC, Chiou SS, Schurman DJ, Woolson ST, Masada MP (1989). A clinical-pathologic-biochemical study of the membrane surrounding loosened and nonloosened total hip arthroplasties. *Clinical Orthopaedics and Related Research*.

[52] Jiranek WA, Machado M, Jasty M (1993). Production of cytokines around loosened cemented acetabular components: analysis with immunohistochemical techniques and in situ hybridization. *Journal of Bone and Joint Surgery A*.

[53] Kusano K, Miyaura C, Inada M (1998). Regulation of matrix metalloproteinases (MMP-2,-3,-9, and -13) by interleukin-1 and interleukin-6 in mouse calvaria: association of MMP induction with bone resorption. *Endocrinology*.

[54] Ishimi Y, Miyaura C, Jin CH (1990). IL-6 is produced by osteoblasts and induces bone resorption. *Journal of Immunology*.

[55] Kurihara N, Bertolini D, Suda T, Akiyama Y, Roodman GD (1990). IL-6 stimulates osteoclast-like multinucleated cell formation in long term human marrow cultures by inducing IL-1 release. *Journal of Immunology*.

[56] Goldring SR, Jasty M, Roelke MS, Rourke CM, Bringhurst FR, Harris WH (1986). Formation of a synovial-like membrane at the bone-cement interface. Its role in bone resorption and implant loosening after total hip replacement. *Arthritis and Rheumatism*.

[57] Appel AM, Sowder WG, Siverhus SW, Hopson CN, Herman JH (1990). Prosthesis-associated pseudomembrane-induced bone resorption. *British Journal of Rheumatology*.

[58] Takagi M, Konttinen YT, Santavirta S (1994). Extracellular matrix metalloproteinases around loose total hip prostheses. *Acta Orthopaedica Scandinavica*.

[59] Fritz EA, Glant TT, Vermes C, Jacobs JJ, Roebuck KA (2006). Chemokine gene activation in human bone marrow-derived osteoblasts following exposure to particulate wear debris. *Journal of Biomedical Materials Research A*.

[60] Huang Z, Ma T, Ren P-G, Smith RL, Goodman SB (2010). Effects of orthopedic polymer particles on chemotaxis of macrophages and mesenchymal stem cells. *Journal of Biomedical Materials Research A*.

[61] Goodman SB, Ma T (2010). Cellular chemotaxis induced by wear particles from joint replacements. *Biomaterials*.

[62] Hukkanen M, Corbett SA, Batten J (1997). Aseptic loosening of total hip replacement. Macrophage expression of inducible nitric oxide synthase and cyclo-oxygenase-2, together with peroxynitrite formation, as a possible mechanism for early prosthesis failure. *Journal of Bone and Joint Surgery B*.

[63] Harris SG, Padilla J, Koumas L, Ray D, Phipps RP (2002). Prostaglandins as modulators of immunity. *Trends in Immunology*.

[64] Steeve KT, Marc P, Sandrine T, Dominique H, Yannick F (2004). IL-6, RANKL, TNF-alpha/IL-1: interrelations in bone resorption pathophysiology. *Cytokine and Growth Factor Reviews*.

[65] Nagase H, Visse R, Murphy G (2006). Structure and function of matrix metalloproteinases and TIMPs. *Cardiovascular Research*.

[66] Leco KJ, Waterhouse P, Sanchez OH (2001). Spontaneous air space enlargement in the lungs of mice lacking tissue inhibitor of metalloproteinases-3 (TIMP-3). *The Journal of Clinical Investigation*.

[67] Fata JE, Leco KJ, Voura EB (2001). Accelerated apoptosis in the Timp-3-deficient mammary gland. *The Journal of Clinical Investigation*.

[68] English JL, Kassiri Z, Koskivirta I (2006). Individual Timp deficiencies differentially impact pro-MMP-2 activation. *The Journal of Biological Chemistry*.

[69] Ray JM, Stetler-Stevenson WG (1994). The role of matrix metalloproteases and their inhibitors in tumour invasion, metastasis and angiogenesis. *European Respiratory Journal*.

[70] Takei I, Takagi M, Santavirta S (2000). Messenger ribonucleic acid expression of 16 matrix metalloproteinases in bone-implant interface tissues of loose artificial hip joints. *Journal of Biomedical Materials Research*.

[71] Sasaki K, Takagi M, Mandelin J (2001). Quantitative analysis of mRNA expression of TIMPs in the periprosthetic interface tissue of loose hips by real-time PCR system. *Journal of Biomedical Materials Research*.

[72] Li T-F, Santavirta S, Virtanen I, Könönen M, Takagi M, Konttinen YT (1999). Increased expression of EMMPRIN in the tissue around loosened hip prostheses. *Acta Orthopaedica Scandinavica*.

[73] Nawrocki B, Polette M, Burlet H, Birembaut P, Adnet J-J (1999). Expression of gelatinase A and its activator MT1-MMP in the inflammatory periprosthetic response to polyethylene. *Journal of Bone and Mineral Research*.

[74] Hembry RM, Bagga MR, Reynolds JJ, Hamblen DL (1995). Stromelysin, gelatinase A and TIMP-1 in prosthetic interface tissue: a role for macrophages in tissue remodelling. *Histopathology*.

[75] Takagi M (1996). Neutral proteinases and their inhibitors in the loosening of total hip prostheses. *Acta orthopaedica Scandinavica. Supplementum*.

[76] Yokohama Y, Matsumoto T, Hirakawa M (1995). Production of matrix metalloproteinases at the bone-implant interface in loose total hip replacements. *Laboratory Investigation*.

[77] Takagi M, Konttinen YT, Lindy O (1994). Gelatinase/Type IV collagenases in the loosening of total hip replacement endoprostheses. *Clinical Orthopaedics and Related Research*.

[78] Takagi M, Konttinen YT, Kemppinen P (1995). Tissue inhibitor of metalloproteinase 1, collagenolytic and gelatinolytic activity in loose hip endoprostheses. *Journal of Rheumatology*.

[79] Santavirta S, Takagi M, Konttinen YT, Sorsa T, Suda A (1996). Inhibitory effect of cephalothin on matrix metalloproteinase activity around loose hip prostheses. *Antimicrobial Agents and Chemotherapy*.

[80] Ishiguro N, Ito T, Kurokouchi K (1996). mRNA expression of matrix metalloproteinases and tissue inhibitors of metalloproteinase in interface tissue around implants in loosening total hip arthroplasty. *Journal of Biomedical Materials Research*.

[81] Strongin AY, Collier I, Bannikov G, Marmer BL, Grant GA, Goldberg GI (1995). Mechanism of cell surface activation of 72-kDa type IV collagenase. Isolation of the activated form of the membrane metalloprotease. *The Journal of Biological Chemistry*.

[82] Syggelos SA, Eleftheriou SC, Giannopoulou E, Panagiotopoulos E, Aletras AJ (2001). Gelatinolytic and collagenolytic activity in periprosthetic tissues from loose hip endoprostheses. *Journal of Rheumatology*.

[83] Goldberg GI, Wilhelm SM, Kronberger A, Bauer EA, Grant GA, Eisen AZ (1986). Human fibroblast collagenase. *The Journal of Biological Chemistry*.

[84] Hasty KA, Jeffrey JJ, Hibbs MS, Welgus HG (1987). The collagen substrate specificity of human neutrophil collagenase. *The Journal of Biological Chemistry*.

[85] Chambers TJ, Darby JA, Fuller K (1985). Mammalian collagenase predisposes bone surfaces to osteoclastic resorption. *Cell and Tissue Research*.

[86] Nordsletten L, Buø L, Takagi M (1996). The plasminogen activation system is upregulated in loosening of total hip prostheses. *Acta Orthopaedica Scandinavica*.

[87] Kazes I, Delarue F, Hagège J (1998). Soluble latent membrane-type 1 matrix metalloprotease secreted by human mesangial cells is activated by urokinase. *Kidney International*.

[88] Carmeliet P, Moons L, Lijnen R (1997). Urokinase-generated plasmin activates matrix metalloproteinases during aneurysm formation. *Nature Genetics*.

[89] Diehl P, Hantke B, Hennig M (2004). Protein expression of MMP-13, uPA, and PAI-1 in pseudocapsular and interface tissue around implants of loose artificial hip joints and in osteoarthritis. *International Journal of Molecular Medicine*.

[90] Nakashima Y, Sun D-H, Maloney WJ, Goodman SB, Schurman DJ, Smith RL (1998). Induction of matrix metalloproteinase expression in human macrophages by orthopaedic particulate debris *in vitro*. *Journal of Bone and Joint Surgery B*.

[91] Laquerriere P, Grandjean-Laquerriere A, Addadi-Rebbah S (2004). MMP-2, MMP-9 and their inhibitors TIMP-2 and TIMP-1 production by human monocytes *in vitro* in the presence of different forms of hydroxyapatite particles. *Biomaterials*.

[92] Ikeda T, Kasai M, Utsuyama M, Hirokawa K (2001). Determination of three isoforms of the receptor activator of nuclear factor-*κ*B ligand and their differential expression in bone and thymus. *Endocrinology*.

[93] Boyle WJ, Simonet WS, Lacey DL (2003). Osteoclast differentiation and activation. *Nature*.

[94] Schwarz EM, Lu AP, Goater JJ (2000). Tumor necrosis factor-*α*/nuclear transcription factor-*κ*B signaling in periprosthetic osteolysis. *Journal of Orthopaedic Research*.

[95] Clohisy JC, Hirayama T, Frazier E, Han S-K, Abu-Amer Y (2004). NF-kB signaling blockade abolishes implant particle-induced osteoclastogenesis. *Journal of Orthopaedic Research*.

[96] Ren W, Li XH, Chen BD, Wooley PH (2004). Erythromycin inhibits wear debris-induced osteoclastogenesis by modulation of murine macrophage NF-*κ*B activity. *Journal of Orthopaedic Research*.

[97] Lamoureux F, Moriceau G, Picarda G, Rousseau J, Trichet V, Rédini F (2010). Regulation of osteoprotegerin pro- or anti-tumoral activity by bone tumor microenvironment. *Biochimica et Biophysica Acta*.

[98] Ulrich-Vinther M, Carmody EE, Goater JJ, Soøballe K, O’Keefe RJ, Schwarz EM (2002). Recombinant adeno-associated virus-mediated osteoprotegerin gene therapy inhibits wear debris-induced osteolysis. *Journal of Bone and Joint Surgery A*.

[99] Jeffrey Goater J, O’Keefe RJ, Rosier RN, Edward Puzas J, Schwarz EM (2002). Efficacy of ex vivo OPG gene therapy in preventing wear debris induced osteolysis. *Journal of Orthopaedic Research*.

[100] Childs LM, Paschalis EP, Xing L (2002). *In vivo* RANK signaling blockade using the receptor activator of NF-*κ*B:Fc effectively prevents and ameliorates wear debris-induced osteolysis via osteoclast depletion without inhibiting osteogenesis. *Journal of Bone and Mineral Research*.

[101] Masui T, Sakano S, Hasegawa Y, Warashina H, Ishiguro N (2005). Expression of inflammatory cytokines, RANKL and OPG induced by titanium, cobalt-chromium and polyethylene particles. *Biomaterials*.

[102] Mandelin J, Li T-F, Liljeström M (2003). Imbalance of RANKL/RANK/OPG system in interface tissue in loosening of total hip replacement. *Journal of Bone and Joint Surgery B*.

[103] Gehrke T, Sers C, Morawietz L (2003). Receptor activator of nuclear factor *κ*B ligand is expressed in resident and inflammatory cells in aseptic and septic prosthesis loosening. *Scandinavian Journal of Rheumatology*.

[104] Haynes DR, Crotti TN, Potter AE (2001). The osteoclastogenic molecules RANKL and RANK are associated with periprosthetic osteolysis. *Journal of Bone and Joint Surgery B*.

[105] Horiki M, Nakase T, Myoui A (2004). Localization of RANKL in osteolytic tissue around a loosened joint prosthesis. *Journal of Bone and Mineral Metabolism*.

[106] Mandelin J, Li T-F, Hukkanen M (2005). Interface tissue fibroblasts from loose total hip replacement prosthesis produce receptor activator of nuclear factor-*κ*B ligand, osteoprotegerin, and cathepsin K. *Journal of Rheumatology*.

[107] Labropoulou VT, Theocharis AD, Symeonidis A, Skandalis SS, Karamanos NK, Kalofonos HP (2011). Pathophysiology and pharmacological targeting of tumor-induced bone disease: current status and emerging therapeutic interventions. *Current Medicinal Chemistry*.

[108] Ishibashi O, Niwa S, Kadoyama K, Inui T (2006). MMP-9 antisense oligodeoxynucleotide exerts an inhibitory effect on osteoclastic bone resorption by suppressing cell migration. *Life Sciences*.

[109] Duivenvoorden WCM, Hirte HW, Singh G (1997). Use of tetracycline as an inhibitor of matrix metalloproteinase activity secreted by human bone-metastasizing cancer cells. *Invasion and Metastasis*.

[110] Franco GCN, Kajiya M, Nakanishi T (2011). Inhibition of matrix metalloproteinase-9 activity by doxycycline ameliorates RANK ligand-induced osteoclast differentiation *in vitro* and *in vivo*. *Experimental Cell Research*.

[111] Blavier L, Delaissé JM (1995). Matrix metalloproteinases are obligatory for the migration of preosteoclasts to the developing marrow cavity of primitive long bones. *Journal of Cell Science*.

[112] Engsig MT, Chen Q-J, Vu TH (2000). Matrix metalloproteinase 9 and vascular endothelial growth factor are essential for osteoclast recruitment into developing long bones. *Journal of Cell Biology*.

[113] Lynch CC, Hikosaka A, Acuff HB (2005). MMP-7 promotes prostate cancer-induced osteolysis via the solubilization of RANKL. *Cancer Cell*.

[114] Wilson TJ, Nannuru KC, Futakuchi M, Sadanandam A, Singh RK (2008). Cathepsin G enhances mammary tumor-induced osteolysis by generating soluble receptor activator of nuclear factor-*κ*B ligand. *Cancer Research*.

[115] Thiolloy S, Halpern J, Holt GE (2009). Osteoclast-derived matrix metalloproteinase-7, but not matrix metalloproteinase-9, contributes to tumor-induced osteolysis. *Cancer Research*.

[116] Standal T, Seidel C, Hjertner Ø (2002). Osteoprotegerin is bound, internalized, and degraded by multiple myeloma cells. *Blood*.

[117] Simonet WS, Lacey DL, Dunstan CR (1997). Osteoprotegerin: a novel secreted protein involved in the regulation of bone density. *Cell*.

[118] Manon-Jensen T, Itoh Y, Couchman JR (2010). Proteoglycans in health and disease: the multiple roles of syndecan shedding. *FEBS Journal*.

[119] Li Q, Park PW, Wilson CL, Parks WC (2002). Matrilysin shedding of syndecan-1 regulates chemokine mobilization and transepithelial efflux of neutrophils in acute lung injury. *Cell*.

[120] Brule S, Charnaux N, Sutton A (2006). The shedding of syndecan-4 and syndecan-1 from HeLa cells and human primary macrophages is accelerated by SDF-1/CXCL12 and mediated by the matrix metalloproteinase-9. *Glycobiology*.

[121] Fears CY, Gladson CL, Woods A (2006). Syndecan-2 is expressed in the microvasculature of gliomas and regulates angiogenic processes in microvascular endothelial cells. *The Journal of Biological Chemistry*.

[122] Endo K, Takino T, Miyamori H (2003). Cleavage of syndecan-1 by membrane type matrix metalloproteinase-1 stimulates cell migration. *The Journal of Biological Chemistry*.

[123] Vermes C, Roebuck KA, Chandrasekaran R, Dobai JG, Jacobs JJ, Glant TT (2000). Particulate wear debris activates protein tyrosine kinases and nuclear factor *κ*B, which down-regulates type I collagen synthesis in human osteoblasts. *Journal of Bone and Mineral Research*.

[124] Luo L, Petit A, Antoniou J (2005). Effect of cobalt and chromium ions on MMP-1, TIMP-1, and TNF-*α* gene expression in human U937 macrophages: a role for tyrosine kinases. *Biomaterials*.

[125] Yan G-R, Xiao C-L, He G-W (2010). Global phosphoproteomic effects of natural tyrosine kinase inhibitor, genistein, on signaling pathways. *Proteomics*.

[126] Stahtea XN, Kousidou OC, Roussidis AE, Tzanakakis GN, Karamanos NK (2008). Small tyrosine kinase inhibitors as key molecules in the expression of metalloproteinases by solid tumors. *Connective Tissue Research*.

[127] Ravindranath MH, Muthugounder S, Presser N, Viswanathan S (2004). Anticancer therapeutic potential of soy isoflavone, genistein. *Advances in Experimental Medicine and Biology*.

[128] Goodsell DS (2002). The molecular perspective: VEGF and angiogenesis. *Oncologist*.

[129] Harry LE, Paleolog EM (2003). From the cradle to the clinic: VEGF in developmental, physiological, and pathological angiogenesis. *Birth Defects Research C*.

[130] Waltenberger J, Claesson-Welsh L, Siegbahn A, Shibuya M, Heldin C-H (1994). Different signal transduction properties of KDR and Flt1, two receptors for vascular endothelial growth factor. *The Journal of Biological Chemistry*.

[131] Kunstfeld R, Hirakawa S, Hong Y-K (2004). Induction of cutaneous delayed-type hypersensitivity reactions in VEGF-A transgenic mice results in chronic skin inflammation associated with persistent lymphatic hyperplasia. *Blood*.

[132] Nakagawa M, Kaneda T, Arakawa T (2000). Vascular endothelial growth factor (VEGF) directly enhances osteoclastic bone resorption and survival of mature osteoclasts. *FEBS Letters*.

[133] Henriksen K, Karsdal M, Delaissé J-M, Engsig MT (2003). RANKL and vascular endothelial growth factor (VEGF) induce osteoclast chemotaxis through an ERK1/2-dependent mechanism. *The Journal of Biological Chemistry*.

[134] Al-Saffar N, Mah JTL, Kadoya Y, Revell PA (1995). Neovascularisation and the induction of cell adhesion molecules in response to degradation products from orthopaedic implants. *Annals of the Rheumatic Diseases*.

[135] Wang JY, Wicklund BH, Gustilo RB, Tsukayama DT (1996). Titanium, chromium and cobalt ions modulate the release of bone-associated cytokines by human monocytes/macrophages *in vitro*. *Biomaterials*.

[136] Niki Y, Matsumoto H, Suda Y (2003). Metal ions induce bone-resorbing cytokine production through the redox pathway in synoviocytes and bone marrow macrophages. *Biomaterials*.

[137] Langholz O, Röckel D, Mauch C (1995). Collagen and collagenase gene expression in three-dimensional collagen lattices are differentially regulated by *α*1*β*1 and *α*2*β*1 integrins. *Journal of Cell Biology*.

[138] Ravanti L, Heino J, López-Otín C, Kaharin VM (1999). Induction of collagenase-3 (MMP-13) expression in human skin fibroblasts by three-dimensional collagen is mediated by p38 mitogen-activated protein kinase. *The Journal of Biological Chemistry*.

[139] Tamaki Y, Takakubo Y, Goto K (2009). Increased expression of toll-like receptors in aseptic loose periprosthetic tissues and septic synovial membranes around total hip implants. *Journal of Rheumatology*.

[140] Gebbia JA, Coleman JL, Benach JL (2004). Selective induction of matrix metalloproteinases by Borrelia burgdorferi via Toll-like receptor 2 in monocytes. *Journal of Infectious Diseases*.

[141] Lisboa RA, Andrade MV, Cunha-Melo JR (2013). Toll-like receptor activation and mechanical force stimulation promote the secretion of matrix metalloproteinases 1, 3 and 10 of human periodontal fibroblasts via p38, JNK and NF-kB. *Archives of Oral Biology*.

[142] Malik MHA, Jury F, Bayat A, Ollier WER, Kay PR (2007). Genetic susceptibility to total hip arthroplasty failure: a preliminary study on the influence of matrix metalloproteinase 1, interleukin 6 polymorphisms and vitamin D receptor. *Annals of the Rheumatic Diseases*.

[143] Ye S (2000). Polymorphism in matrix metalloproteinase gene promoters: implication in regulation of gene expression and susceptibility of various diseases. *Matrix Biology*.

[144] Benbow U, Rutter JL, Lowrey CH, Brinckerhoff CE (1999). Transcriptional repression of the human collagenase-1 (MMP-1) gene in MDA231 breast cancer cells by all-trans-retinoic acid requires distal regions of the promoter. *British Journal of Cancer*.

[145] Bramhall SR, Rosemurgy A, Brown PD, Bowry C, Buckles JAC (2001). Marimastat as first-line therapy for patients with unresectable pancreatic cancer: a randomized trial. *Journal of Clinical Oncology*.

[146] Ortiz-Lazareno PC, Hernandez-Flores G, Dominguez-Rodriguez JR (2008). MG132 proteasome inhibitor modulates proinflammatory cytokines production and expression of their receptors in U937 cells: involvement of nuclear factor-*κ*B and activator protein-1. *Immunology*.

[147] Mao X, Pan X, Cheng T, Zhang X (2012). Inhibition of titanium particle-induced inflammation by the proteasome inhibitor bortezomib in murine macrophage-like RAW 264.7 cells. *Inflammation*.

[148] Krakauer T (2004). Molecular therapeutic targets in inflammation: cyclooxygenase and NF-*κ*B. *Current Drug Targets*.

[149] Goffin L, Seguin-Estévez Q, Alvarez M, Reith W, Chizzolini C (2010). Transcriptional regulation of matrix metalloproteinase-1 and collagen 1A2 explains the anti-fibrotic effect exerted by proteasome inhibition in human dermal fibroblasts. *Arthritis Research and Therapy*.

[150] Smallie T, Ricchetti G, Horwood NJ, Feldmann M, Clark AR, Williams LM (2010). IL-10 inhibits transcription elongation of the human TNF gene in primary macrophages. *The Journal of Experimental Medicine*.

[151] Lentsch AB, Shanley TP, Sarma V, Ward PA (1997). *In vivo* suppression of NF-*κ*B and preservation of I*κ*B*α* by interleukin- 10 and interleukin-13. *The Journal of Clinical Investigation*.

[152] Giuliani N, Morandi F, Tagliaferri S (2007). The proteasome inhibitor bortezomib affects osteoblast differentiation *in vitro* and *in vivo* in multiple myeloma patients. *Blood*.

[153] Terpos E, Heath DJ, Rahemtulla A (2006). Bortezomib reduces serum dickkopf-1 and receptor activator of nuclear factor-*κ*B ligand concentrations and normalises indices of bone remodelling in patients with relapsed multiple myeloma. *British Journal of Haematology*.

[154] von Metzler I, Krebbel H, Hecht M (2007). Bortezomib inhibits human osteoclastogenesis. *Leukemia*.

[155] Palombella VJ, Rando OJ, Goldberg AL, Maniatis T (1994). The ubiquitin-proteasome pathway is required for processing the NF-*κ*B1 precursor protein and the activation of NF-*κ*B. *Cell*.

[156] Reinstein E, Ciechanover A (2006). Narrative review: potein degradation and human diseases: the ubiquitin connection. *Annals of Internal Medicine*.

[157] Skandalis SS, Aletras AJ, Gialeli C (2012). Targeting the tumor proteasome as a mechanism to control the synthesis and bioactivity of matrix macromolecules. *Current Molecular Medicine*.

[158] Wang ML, Hauschka PV, Tuan RS, Steinbeck MJ (2002). Exposure to particles stimulates superoxide production by human THP-1 macrophages and A vian HD-11EM osteoclasts activated by tumor necrosis factor-*α* and PMA. *Journal of Arthroplasty*.

[159] Wu H-M, Chi K-H, Lin W-W (2002). Proteasome inhibitors stimulate activator protein-1 pathway via reactive oxygen species production. *FEBS Letters*.

[160] Catalgol B, Ziaja I, Breusing N (2009). The proteasome is an integral part of solar ultraviolet a radiation-induced gene expression. *The Journal of Biological Chemistry*.

[161] Fineschi S, Reith W, Guerne PA, Dayer J-M, Chizzolini C (2006). Proteasome blockade exerts an antifibrotic activity by coordinately down-regulating type I collagen and tissue inhibitor of metalloproteinase-1 and up-regulating metalloproteinase-1 production in human dermal fibroblasts. *FASEB Journal*.

[162] Kim H-J, Barajas B, Wang M, Nel AE (2008). Nrf2 activation by sulforaphane restores the age-related decrease of TH1 immunity: role of dendritic cells. *Journal of Allergy and Clinical Immunology*.

[163] Sepp-Lorenzino L, Ma Z, Lebwohl DE, Vinitsky A, Rosen N (1995). Herbimycin A induces the 20 S proteasome- and ubiquitin-dependent degradation of receptor tyrosine kinases. *The Journal of Biological Chemistry*.

[164] Markel DC, Zhang R, Shi T, Hawkins M, Ren W (2009). Inhibitory effects of erythromycin on wear debris-induced VEGF/Flt-1 gene production and osteolysis. *Inflammation Research*.

[165] Millett PJ, Allen MJ, Bostrom MPG (2002). Effects of alendronate on particle-induced osteolysis in a rat model. *Journal of Bone and Joint Surgery A*.

[166] von Knoch M, Wedemeyer C, Pingsmann A (2005). The decrease of particle-induced osteolysis after a single dose of bisphosphonate. *Biomaterials*.

[167] Dedes PG, Gialeli C, Tsonis A (2012). Expression of matrix macromolecules and functional properties of breast cancer cells are modulated by the bisphosphonate zoledronic acid. *Biochimica et Biophysica Acta*.

[168] Ito A, Nose T, Takahashi S, Mori Y (1995). Cyclooxygenase inhibitors augment the production of pro-matrix metalloproteinase 9 (progelatinase B) in rabbit articular chondrocytes. *FEBS Letters*.

[169] DiBattista JA, Martel-Pelletier J, Fujimoto N, Obata K, Zafarullah M, Pelletier J-P (1994). Prostaglandins E2 and E1 inhibit cytokine-induced metalloprotease expression in human synovial fibroblasts: mediation by cyclic-AMP signalling pathway. *Laboratory Investigation*.

[170] Takahashi S, Inoue T, Higaki M, Mizushima Y (1997). Cyclooxygenase inhibitors enhance the production of tissue inhibitor-1 of metalloproteinases (TIMP-1) and pro-matrix metalloproteinase 1 (proMMP-1) in human rheumatoid synovial fibroblasts. *Inflammation Research*.

[171] Lin SK, Wang CC, Huang S (2001). Induction of dental pulp fibroblast matrix metalloproteinase-1 and tissue inhibitor of metalloproteinase-1 gene expression by interleukin-1alpha and tumor necrosis factor-alpha through a prostaglandin-dependent pathway. *Journal of Endodontics*.

[172] Yamazaki R, Kawai S, Mizushima Y (2000). A major metabolite of aceclofenac, 4’-hydroxy aceclofenac, suppresses the production of interstitial pro-collagenase/proMMP-1 and pro-stromelysin- 1/proMMP-3 by human rheumatoid synovial cells. *Inflammation Research*.

[173] Akimoto H, Yamazaki R, Hashimoto S, Sato T, Ito A (2000). 4′-Hydroxy aceclofenac suppresses the interleukin-1-induced production of promatrix metalloproteinases and release of sulfated-glycosaminoglycans from rabbit articular chondrocytes. *European Journal of Pharmacology*.

[174] Syggelos SA, Giannopoulou E, Gouvousis PA, Andonopoulos AP, Aletras AJ, Panagiotopoulos E (2007). *In vitro* effects of non-steroidal anti-inflammatory drugs on cytokine, prostanoid and matrix metalloproteinase production by interface membranes from loose hip or knee endoprostheses. *Osteoarthritis and Cartilage*.

[175] Ishimi Y, Miyaura C, Jin CH (1990). IL-6 is produced by osteoblasts and induces bone resorption. *Journal of Immunology*.

[176] Kurihara N, Bertolini D, Suda T, Akiyama Y, Roodman GD (1990). IL-6 stimulates osteoclast-like multinucleated cell formation in long term human marrow cultures by inducing IL-1 release. *Journal of Immunology*.

[177] Lerner UH, Ohlin A (1993). Tumor necrosis factors *α* and *β* can stimulate bone resorption in cultured mouse calvariae by a prostaglandin-independent mechanism. *Journal of Bone and Mineral Research*.

[178] Herman JH, Sowder WG, Hess EV (1994). Nonsteroidal antiinflammatory drug modulation of prosthesis pseudomembrane induced bone resorption. *Journal of Rheumatology*.

